# Dyeing of Tussah Silk with Reactive Dyes: Dye Selection, Dyeing Conditions, Dye Fixation Characteristics, and Comparison with Mulberry Silk

**DOI:** 10.3390/molecules29051151

**Published:** 2024-03-05

**Authors:** Yingjie Yu, Rencheng Tang

**Affiliations:** 1 College of Textile and Clothing Engineering, North Campus, Soochow University, Suzhou 215021, China; 2 China National Textile and Apparel Council Key Laboratory of Natural Dyes, Dushuhu Campus, Soochow University, Suzhou 215123, China; 3 Jiangsu Engineering Research Center of Textile Dyeing and Printing for Energy Conservation, Discharge Reduction and Cleaner Production (ERC), Dushuhu Campus, Soochow University, Suzhou 215123, China; 4 National Engineering Laboratory for Modern Silk, Dushuhu Campus, Soochow University, Suzhou 215123, China

**Keywords:** tussah silk, dyeing, reactive dyes, exhaustion, fixation, color fastness

## Abstract

Tussah silk is one of the most widely used wild silks. It is usually dyed with acid dyes, despite the shortcoming of poor wet fastness. Reactive dyeing is a good solution to this problem. In our work, sulfatoethylsulfone (SES), sulfatoethylsulfone/monochlorotriazine (SES/MCT), monochlorotriazine (MCT), and bis(monochlorotriazine) (Bis(MCT)) dyes were used to dye tussah silk. All of these dyes showed lower exhaustion and fixation on tussah silk than on mulberry silk under alkaline conditions. Among them, SES dyes were more applicable, with a fixation of 70–85% (at 4%owf dye) at 90 °C when using sodium bicarbonate as an alkali. SES dyes also showed a rapid fixation speed. The dyeing of tussah silk required lower sodium bicarbonate dosage, the use of more neutral electrolytes, and a higher dye quantity to achieve deep effects compared to mulberry silk. Dyed tussah silk displayed lower apparent color depth and brilliance than dyed mulberry silk. The neutral boiling dyeing of tussah silk with SES dyes exhibited higher exhaustion, higher fixation (82–92% at 4%owf dye), and a slower fixation speed compared with alkaline dyeing. Furthermore, in this dyeing method, SES dyes showed higher and more efficient fixation on tussah silk than on mulberry silk. All dyed tussah silk had excellent color fastness to soaping.

## 1. Introduction

Tussah silk is one of the most important wild silks; it is also a protein fiber, like mulberry silk and wool. In theory, protein fibers have similar dyeing properties, e.g., good dyeability with acid dyes. However, there are significant differences between tussah and mulberry silks in terms of their appearance, color, amino acid composition, morphological structure, crystalline structure, and pore structure [1], which lead to some differences between the dyeing properties of the two silk fibers. Existing dyeing methods for tussah silk face two big problems: poor color fastness to washing and great difficulty in reaching satisfactory color depth [2,3]. The issue of poor fastness is primarily related to the dyes used. Silk products are commonly dyed with acid dyes, 1:2 metal complex dyes, and sometimes, direct dyes [4]. However, these dyes, especially acid and direct dyes, suffer from poor wet color fastness [3,5,6]. Our study revealed that dyes displayed a higher extent of desorption from tussah silk than from mulberry silk during washing, resulting in poor fastness. An early literature review reported that obtaining dark colors (e.g., dark brown, navy, and black) on dyed tussah silk is difficult, likely owing to its flat cross-section. Additionally, tussah silk requires a higher quantity of dyes to achieve a similar color depth as mulberry silk [2]. Reactive dyes can react covalently with protein fibers. Therefore, reactive dyeing is the best solution to the problem of the poor washing fastness of dyed tussah silk.

The dyeing of silk with reactive dyes has the advantages of bright colors, a wide spectrum of colors, and good wet fastness [7,8]. Compared with the reactive dyeing of mulberry silk, there are few research reports on the reactive dyeing of tussah silk. In previous studies, sulfatoethylsulfone/monochlorotriazine (SES/MCT), bis(monochlorotriazine) (Bis(MCT)), mononicotinic acid triazine (MNT), and dichlorotriazine (DCT) dyes were used to dye tussah silk [9,10,11,12,13,14]. However, there are extremely few comparative studies on the dyeing of tussah and mulberry silks with reactive dyes, and there are no reports on the selection of reactive dyes for the dyeing of tussah silk. In addition, previously reported dyeing conditions of reactive dyes for tussah silk had great differences, and the fixation of reactive dyes used was low, ranging mostly from 60% to 70% (Table 1) [9,10,11,12,13,14], which was obviously lower than that observed for the dyeing of mulberry silk [15,16,17,18,19,20]. Such low percentages of dye fixation lead to low dye utilization, high dyeing costs, and an increased burden of washing and soaping. Therefore, it is necessary to carry out systematic and in-depth research on the selection of reactive dyes, control over dyeing conditions, and mechanisms of dye fixation for the dyeing of tussah silk.

Tussah silk contains abundant functional groups that can react with reactive dyes, i.e., typically amine groups in alkaline amino acids and hydroxyl groups in hydroxyl amino acids. As shown in Table 2, the hydroxyl amino acid content of tussah silk is slightly lower than that of mulberry silk, while both silks have roughly equal tyrosine contents. The content of tyrosine in tussah silk is as high as 10.6%wt, and tyrosine is mainly distributed in the amorphous region of the fibers [21]. Tyrosine plays an important role in the reactive dyeing of silk [8]. However, the phenolic hydroxyl group in tyrosine must be ionized before tyrosine reacts with reactive dyes, and consequently, the use of an alkaline dyebath is favorable to its reaction. The alkaline amino acid content of tussah silk is 3.88 times higher than that of mulberry silk. The arginine content of tussah silk is as high as 5.4%wt, which is significantly higher than that of mulberry silk. The reaction between the amino group in arginine and reactive dyes (e.g., MCT group) does not require alkaline conditions. Tussah silk has significantly higher tyrosine content than wool, but its arginine content is half that of wool. Wool is usually dyed with reactive dyes under weakly acidic conditions, followed by weakly alkaline soaping [22], while weakly alkaline fixation conditions are mostly employed for the reactive dyeing of mulberry silk [15,16,17,18,19,20]. Reactive dyes mainly react with the alkaline amino acids in wool [23], while the reaction can occur at the amine and hydroxyl groups in amino acids for the reactive dyeing of silk [8]. Can the reactive dyeing of tussah silk be carried out under weakly acidic conditions, or would it be better if neutral electrolytes are first used to promote the adsorption of dyes and then weakly alkaline conditions are used to fix the dyes onto the fibers? These questions are very interesting and worth studying. In this regard, tussah silk is more resistant to alkali compounds than mulberry silk, which is advantageous in reactive dyeing under alkaline conditions [2].

In our work, sulfatoethylsulfone (SES), SES/MCT, MCT, and Bis(MCT) dyes were used to dye tussah silk. The color index (C.I.), trade name, chromophore, molecular mass, and number of sulfonate groups of dyes are listed in Table 3, and the chemical structures of the dyes are shown in Figure S1. In our preliminary experiments, four types of dyes showed low fixation on tussah silk under weakly acidic conditions due to the main contribution of electrostatic attraction between dyes and fibers to the uptake of dyes. Afterward, the neutral dyeing and alkaline fixing process (called “alkaline dyeing”) was employed. The results showed that when the dyeing was conducted at 90 °C in the case of sodium bicarbonate, SES dyes exhibited a high degree of fixation on tussah silk. Therefore, a systematic study was carried out in terms of the alkali dosage, neutral electrolyte dosage, liquor ratio, exhaustion and fixation kinetics of dyes, build-up property of dyes, soaping color fastness, and dye fixation mechanism. In addition, neutral boiling dyeing with the use of a neutral electrolyte as a dyeing accelerator and without the use of an alkali was further tested, and the feasibility of this process was discussed. In this study, we also performed a comparison of the reactive dyeing properties of tussah and mulberry silks. The present work may serve as a technical foundation for the reactive dyeing of tussah silk in terms of dye selection, dyeing condition control, and the improvement of wet color fastness.

## 2. Results and Discussion

### 2.1. Selection of Reactive Dyes for the Dyeing of Tussah Silk

#### 2.1.1. SES/MCT Dyes: Effect of Temperature and Alkali

The reactive dyeing of mulberry silk is generally carried out under alkaline conditions [15,16,17,18,19,20,26,27]. Considering this, the dyeing of tussah silk with SES, MCT, SES/MCT, and Bis(MCT) dyes at various temperatures and in the presence of sodium carbonate and sodium bicarbonate was studied. In the meantime, the dyeing of tussah and mulberry silks was compared. Considering that SES/MCT dyes are widely used among all reactive dyes and are very suitable for the dyeing of mulberry silk [15,18,19,28], trichromatic SES/MCT dyes were first tested. Figure S2 shows that when sodium carbonate was used as an alkaline agent, the exhaustion and fixation of three SES/MCT dyes on tussah silk were very low at various temperatures, reaching the maximum values at 60 °C (exhaustion 27–32% and fixation 22–28%). However, the maximum exhaustion (71–76%) and fixation (69–72%) of three dyes on mulberry silk occurring at 60 °C were markedly higher than those on tussah silk. The exhaustion and fixation of dyes decreased significantly at 70 and 80 °C due to the hydrolysis of the dyes.

When sodium bicarbonate was used as an alkaline agent, the exhaustion and fixation of SES/MCT dyes on tussah and mulberry silks increased with increasing temperature and were higher than those when sodium carbonate was used because of the reduction in the hydrolysis of dyes. The greatest exhaustion and fixation of the three dyes on mulberry silk occurring at 80–90 °C were 79–82% and 75–80%, respectively. For the dyeing of tussah silk, the highest exhaustion and fixation of dyes occurred at 90 °C, but the values were 49–52% and 43–48%, respectively, which were obviously lower than those observed for mulberry silk. Despite a significant improvement in the exhaustion and fixation of dyes in the presence of sodium bicarbonate, such low fixation does not meet the requirements for current dyeing process due to the low rate of dye utilization and the high cost of dyes.

#### 2.1.2. SES, MCT, and Bis(MCT) Dyes: Effect of Temperature and Alkali

The above experiments revealed that the exhaustion and fixation of SES/MCT dyes on tussah silk were relatively low. Therefore, it is necessary to test the application of other reactive dyes under alkaline fixation conditions at various temperatures. Herein, red SES, MCT, and Bis(MCT) dyes were selected as representatives for this purpose.

As shown in Figure S3, when sodium carbonate was used as an alkaline agent, the exhaustion and fixation of red SES, MCT, and Bis(MCT) dyes on tussah silk were all low, and all of the fixation values were below 50%. For the dyeing of tussah silk, Red 180 (SES dye), Red 24 (MCT dye), and Red 141 (Bis(MCT)) dye) displayed the maximum fixation, which was 47.8% at 60 °C, 48.0% at 70 °C, and 12.2% at 80 °C, respectively, while the maximum fixation of Red 180, Red 24, and Red 141 was 73% at 50 °C, 73% at 60 °C, and 50% at 70 °C, respectively, for the dyeing of mulberry silk. Like SES/MCT dyes, SES, MCT, and Bis(MCT) dyes also showed lower exhaustion and fixation on tussah silk than on mulberry silk. Thus, the use of sodium carbonate as a fixing agent for dyeing with SES, MCT, and Bis(MCT) dyes is not a feasible approach.

When sodium bicarbonate was used as an alkaline agent, the exhaustion and fixation of the three red dyes increased with increasing temperature and were higher than those when sodium bicarbonate was used as an alkaline agent due to the reduction in the hydrolysis of the dyes. Overall, the maximum exhaustion and fixation occurred at 80–90 °C for tussah and mulberry silks. For the dyeing of tussah silk, Red 141 (Bis(MCT) dye) showed a very low fixation at 90 °C, while Red 24 (MCT dye) had the highest fixation (51%) at 90 °C; meanwhile, the fixation of Red 180 approached 70% at 80–90 °C. This test indicated that SES dyes are likely to be suitable for the dyeing of tussah silk.

#### 2.1.3. Dyeing Results of Various Reactive Dyes

**Figure 1:**
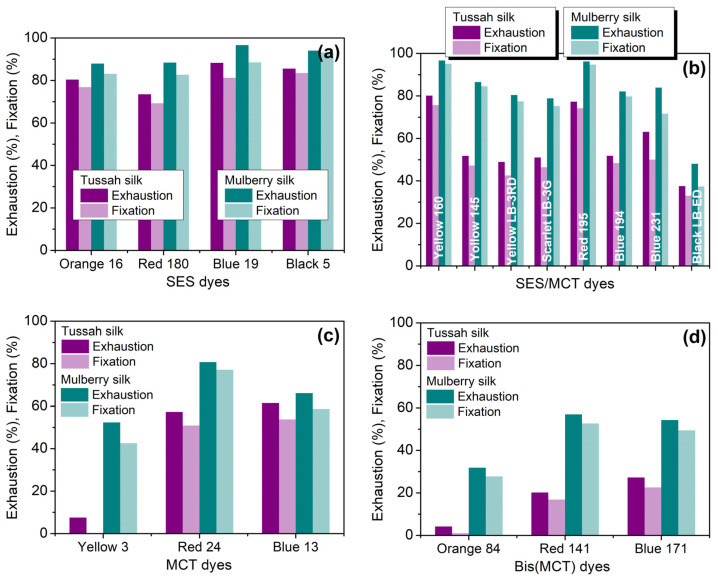
Exhaustion and fixation of SES (**a**), SES/MCT (**b**), MCT (**c**), and Bis(MCT) (**d**) dyes on tussah and mulberry silks dyed at 90 °C using NaHCO_3_ as a fixing agent (3%owf dye, 60 g/L Na_2_SO_4_, and 3 g/L NaHCO_3_).

Taking into account the effects of temperature and alkali depicted in Figures S2 and S3, the use of sodium bicarbonate as an alkaline agent and dyeing at 90 °C are suitable for the dyeing of tussah silk. Under such conditions, the exhaustion and fixation of four SES dyes, eight SES/MCT dyes, three MCT dyes, and three Bis(MCT) dyes on tussah and mulberry silks were measured; the results are shown in Figure 1.

The exhaustion and fixation of SES dyes on tussah silk ranged from 74% to 88% and from 70% to 84%, with an average of 82.0% and 77.7%, respectively, while their fixation on mulberry silk ranged from 82% to 93%, with an average of 86.9%. SES/MCT dyes exhibited significant differences in exhaustion and fixation. The fixation of SES/MCT dyes on tussah silk ranged from 33% to 76%, with an average of 52.1%, while their fixation on mulberry silk was between 37% and 95%, with an average of 76.9%. Among them, Yellow 160 and Red 195 had high fixations of 75.6% and 74.1% on tussah silk, respectively. Among MCT dyes, Yellow 3 could not be readily fixed on tussah silk, and two other dyes had an average fixation of 52.3%, while the fixation of three dyes on mulberry silk ranged from 42% to 77%, with an average of 59.4%. All three Bis(MCT) dyes had a very low fixation on tussah silk, while their fixation on mulberry silk ranged from 27% to 53%, with an average of 43.2%.

Various types of reactive dyes exhibited great changes in exhaustion and fixation, which could be caused by many factors. The strong repulsive forces between dyes and tussah silk and the reactivity of dyes are important factors. The content of anionic acidic amino acids (aspartic acid and glutamic acid) in tussah silk is more than twice that of mulberry silk [24], indicating that tussah silk is more negatively charged, leading to stronger repulsive forces between dyes and tussah silk. Among SES/MCT dyes, Yellow 145, Red 195, and Blue 194 contain three to four sulfonate groups and one SES group, which are all anionic groups. The strong repulsive forces between dyes and fibers lead to a low dye exhaustion, which results in a parallel decrease in dye fixation. Three Bis(MCT) dyes contain more sulfonate groups (six to eight), resulting in very low exhaustion and fixation. In terms of the reactivity of dyes, the reactivity of the MCT group is lower than that of the SES group, which can also lead to the low fixation of MCT and Bis(MCT) dyes. SES dyes have fewer sulfonate groups compared with other types of dyes, and the SES group has a high reactivity, both of which enable them to have high exhaustion and fixation.

From the above experiments, it can be seen that among the dyes tested in this study, all of the four SES dyes showed high exhaustion and fixation on tussah silk, with a fixation efficiency of 90% or more. This indicates that SES dyes on tussah silk have a high rate of reaction, which can reduce the burden of washing and soaping after dyeing and result in high color fastness to soaping.

### 2.2. Dyeing of Tussah Silk with SES Dyes under Alkaline Conditions

The above experiments demonstrate that SES dyes have high exhaustion and fixation on tussah silk. Therefore, the dyeing properties of SES dyes under alkaline fixation conditions with sodium bicarbonate as an alkaline agent were further studied.

#### 2.2.1. Effect of Neutral Electrolyte Dosage

The content of anionic acidic amino acids (aspartic acid and glutamic acid) in tussah silk is more than double that in mulberry silk [24]. This leads to great electrostatic repulsion between reactive dyes and tussah silk; therefore, more inorganic salts should be used to accelerate the uptake of reactive dyes. A previous study revealed that the uptake of one SES/MCT dye (Red M-3BE) by tussah silk was more sensitive to neutral electrolytes than that by mulberry silk [9].

**Figure 2:**
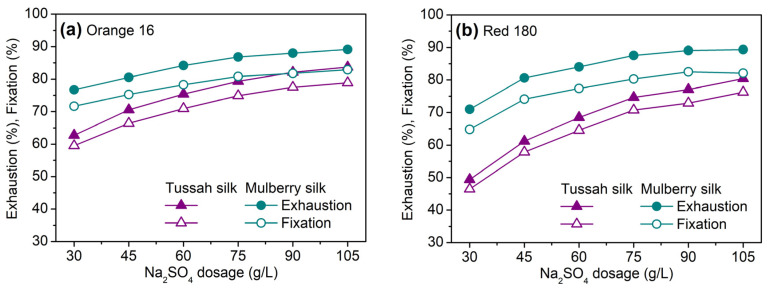
Exhaustion and fixation of Orange 16 (**a**) and Red 180 (**b**) on tussah and mulberry silks dyed at 90 °C and various dosages of Na_2_SO_4_ using NaHCO_3_ as a fixing agent (5%owf dye, 30–105 g/L Na_2_SO_4_, and 3 g/L NaHCO_3_).

In our neutral electrolyte dosage test, Orange 16 with one sulfonate group and Red 180 with three sulfonate groups were used to dye tussah and mulberry silks. The test results are shown in Figure 2. As shown, the dyeing of tussah silk with two SES dyes was more sensitive to neutral electrolytes compared to mulberry silk. With an increase in neutral electrolyte dosage, the exhaustion and fixation of dyes on tussah silk displayed a high increment. Furthermore, as the dosage of neutral electrolytes increased, the difference in the exhaustion and fixation of dyes between tussah and mulberry silks decreased. This test indicated that the dyeing of tussah silk with SES dyes requires a higher quantity of neutral salts compared to mulberry silk to reach high exhaustion and fixation. However, it is well known that a high concentration of neutral salts may lead to dye aggregation and uneven dyeing. In this case, the portion-wise addition of neutral salts can efficiently prevent uneven dyeing by avoiding an excessive uptake speed of dyes.

Red 180 bearing three sulfonate groups was more sensitive to neutral electrolyte dosage than Orange 16 bearing one sulfonate group and needed a higher dosage of neutral electrolytes to accelerate its uptake and fixation, especially when it was used with tussah silk.

#### 2.2.2. Effect of Sodium Bicarbonate Dosage

**Figure 3:**
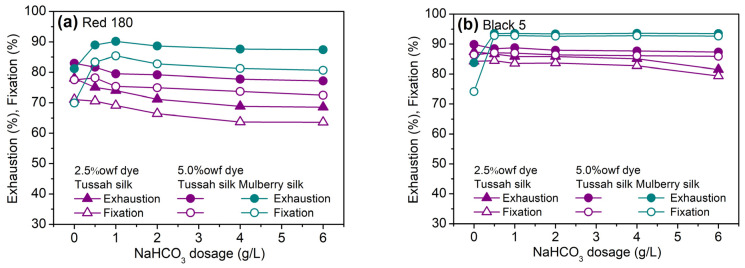
Exhaustion and fixation of Red 180 (**a**) and Black 5 (**b**) on tussah and mulberry silks dyed at 90 °C and various dosages of NaHCO_3_ (60 g/L Na_2_SO_4_ for 2.5%owf dye, 90 g/L Na_2_SO_4_ for 5.0%owf dye, and 0–6 g/L NaHCO_3_).

The above experiments demonstrate that sodium bicarbonate is a more suitable alkaline agent for the reactive dyeing of tussah silk at 90 °C in comparison with sodium carbonate. Furthermore, the effect of sodium bicarbonate dosage on the exhaustion and fixation of SES dyes on tussah and mulberry silks was discussed. In this regard, two SES dyes, Red 180 (SES) and Black 5 (Bis(SES)), were used. In general, the exhaustion and fixation of reactive dyes decreased with an increase in the dosage of reactive dyes. However, in Figure 3, the exhaustion and fixation at 5.0% dye dosage were higher than those at 2.5% dye dosage, which can be explained by different amounts of sodium sulfate at two dye dosages.

As shown in Figure 3, the dosage of sodium bicarbonate had different effects on the dyeing of tussah and mulberry silks. For the dyeing of mulberry silk with 5.0% SES dyes, the exhaustion and fixation of Red 180 increased first and then decreased slightly with increasing sodium bicarbonate dosage, while the exhaustion and fixation of Black 5 reached the maximum at 0.5–1 g/L sodium bicarbonate and then did not undergo any further significant changes. A sodium bicarbonate dosage of 1 g/L could ensure the highest exhaustion and fixation of dyes on mulberry silk, which is consistent with our previously reported result for the dyeing of mulberry silk fabric with SES/MCT dyes [28]. However, the dyeing of tussah silk showed less dependence on sodium bicarbonate dosage compared to mulberry silk and only needed 0–0.5 g/L sodium bicarbonate at the present constant temperature. The low sensitivity of the dyeing of tussah silk with SES dyes to sodium bicarbonate dosage is an advantage for controlling color depth and color difference in the dyeing processing.

**Figure 4:**
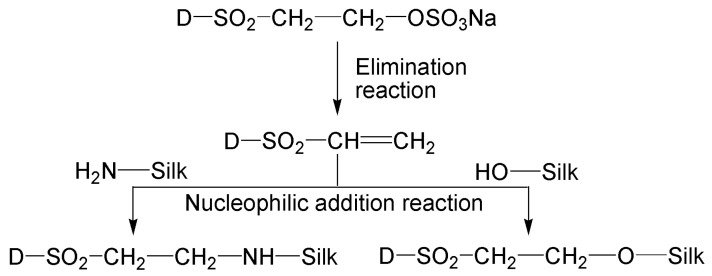
Schematic diagram of the mechanism of reaction between SES dye and tussah silk.

It is worth pointing out that in the absence of sodium bicarbonate, two SES dyes exhibited obviously higher exhaustion and fixation on tussah silk than on mulberry silk. This phenomenon is closely associated with the amino acid composition of tussah silk and the reaction characteristics of SES dyes. It is well known that only after the SES moiety of SES dyes is converted to the reactive vinylsulfone (VS) form can the dyes react with the amine and hydroxyl groups in protein fiber. The mechanism of reaction between SES dye and tussah silk is depicted in Figure 4. An increase in pH, temperature, and time can accelerate the conversion of SES to vs. [22,29]. The high fixation of two SES dyes on tussah and mulberry silks at 90 °C in the absence of alkali demonstrates that the SES moiety can be converted to the vs. form which further reacts with silk. On the other hand, in terms of the content of reactive amino acids in silk fibroin, the content of tyrosine in tussah silk (10.60%wt) is slightly lower than that in mulberry silk (11.29%wt), while the content of basic amino acids in tussah silk (7.13%wt) is significantly higher than that in mulberry silk (1.84%wt) [24]. In particular, the content of arginine in tussah silk is as high as 5.41%wt, in comparison with 0.98%wt in mulberry silk [24]. Therefore, the higher content of basic amino acids in tussah silk causes a higher fixation of SES dyes on tussah silk in the absence of an alkali.

Although the present test at a constant temperature of 90 °C displayed a high fixation of Red 180 and Black 5 on tussah silk, the subsequent experiments revealed the necessity of a small quantity of sodium bicarbonate for fixation efficiency, time-saving, and applicability to more SES dyes, as will be discussed later.

#### 2.2.3. Exhaustion and Fixation Rate

The exhaustion and fixation rate curves, or dyeing kinetics, are an important guideline with which to evaluate the dyeing properties of reactive dyes and can provide meaningful references for determining dyeing conditions and controlling dyeing processes. In the present study, the dyeing of tussah and mulberry silks with Red 180 (SES dye) was taken as an example, and the temperature-rising dyeing procedure was used. When the temperature increased to 90 °C, two fixing methods, i.e., alkaline fixation (addition of sodium bicarbonate) and neutral fixation, were employed.

**Figure 5:**
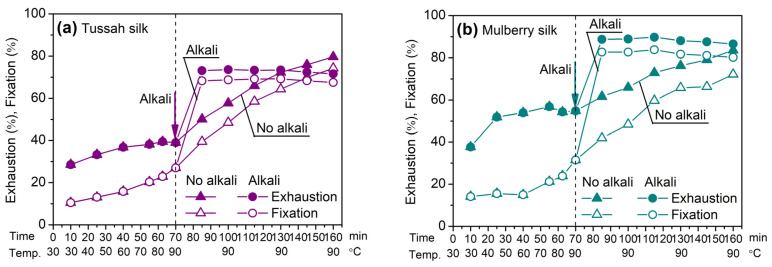
Exhaustion and fixation rates of Red 180 on tussah (**a**) and mulberry (**b**) silks with the temperature-rising dyeing procedure (3%owf dye, 80 g/L Na_2_SO_4_, and 0 or 2 g/L NaHCO_3_).

Figure 5 shows that in the temperature-rising process, the exhaustion of Red 180 on tussah and mulberry silks was low. Moreover, the exhaustion on tussah silk was lower than that on mulberry silk, which is likely because tussah silk has a smaller surface area and a lower swelling extent than mulberry silk [30,31,32,33]. Additionally, during this period, Red 180 had a certain extent of fixation on the two silks. This is related to the conversion of the SES moiety of some dye molecules to the vs. form which can react with amine groups in silk fibroin.

In the process of temperature holding at 90 °C, the addition of sodium bicarbonate led to a rapid increase in the exhaustion and fixation of dyes owing to the rapid conversion of the SES group to the vs. group. Under such an alkaline condition, reactive dyes can react with amine groups and ionized hydroxyl groups in silk fibroin. However, in the absence of an alkali, the exhaustion and fixation of dyes showed a slow increase in dyeing time; this was related to the slow rate of the conversion of the SES group to the VS group at 90 °C and in a neutral medium.

When the temperature was kept at 90 °C, the dye fixation in an alkaline medium was significantly higher than that in an alkali-free medium. However, the difference in dye fixation between alkaline and alkali-free dyeing gradually decreased as the dyeing time was prolonged, and moreover, such a difference for the dyeing of tussah silk was smaller than that for the dyeing of mulberry silk. Very interestingly, for the dyeing of tussah silk, Red 180 exhibited higher final fixation in an alkali-free medium than in an alkaline medium after the dyeing had been conducted for a long time, while such a change did not occur for the dyeing of mulberry silk. These phenomena are related to the higher basic amino acid content of tussah silk compared with mulberry silk. In the absence of an alkali, the reaction of amine groups in silk with VS groups in dyes makes a prominent contribution to total dye fixation because of the low ionization extent of hydroxyl groups in silk.

The present test revealed the rapid fixation of SES dyes on tussah silk after the addition of alkali, which would have a significant impact on the color levelness of fibers. Thus, control of alkali addition (e.g., portion-wise addition) becomes a key factor for level dyeing. If a neutral fixation condition is used with no addition of alkali, the color levelness of fibers is good because the slow reaction between dyes and tussah silk occurs, but a longer dyeing time is necessary for a high fixation to be achieved. Here, it must be pointed out that the subsequent experiment (see Table 4) revealed that the fixation of Orange 16, Blue 19, and Black 5 on tussah silk at 90 °C and under alkaline fixation conditions was higher than that at 90 °C and under a neutral fixation condition for a long dyeing time. Furthermore, SES dyes exhibited higher fixation efficiency under alkaline fixation conditions, which helped to reduce the burden of washing and soaping after dyeing. Therefore, in the case of dyeing at 90 °C, it would be better that the fixation of SES dyes be carried out in an alkaline medium.

#### 2.2.4. Build-Up Property

Previous reviews pointed out that when the same apparent color depth was expected, tussah silk required a greater quantity of acid dyes than mulberry silk, probably due to its flat cross-section [2,34]. Evidently, the build-up ability of reactive dyes is also important for the deep dyeing of tussah silk. Therefore, in the present study, the build-up ability of SES dyes on tussah silk was discussed in terms of the amount of exhaustion, fixation, quantity of fixation, and apparent color depth (K/S). Additionally, the hue angle and color chromaticity of dyed tussah silk were also considered. In this regard, Orange 16, Red 180, and Black 5 (Black 5, Bis(SES)), at various dosages, were used in the dyeing of tussah and mulberry silks in the temperature-rising procedure, and at the stage of temperature holding at 90 °C, sodium bicarbonate was used as an alkaline agent.

**Figure 6:**
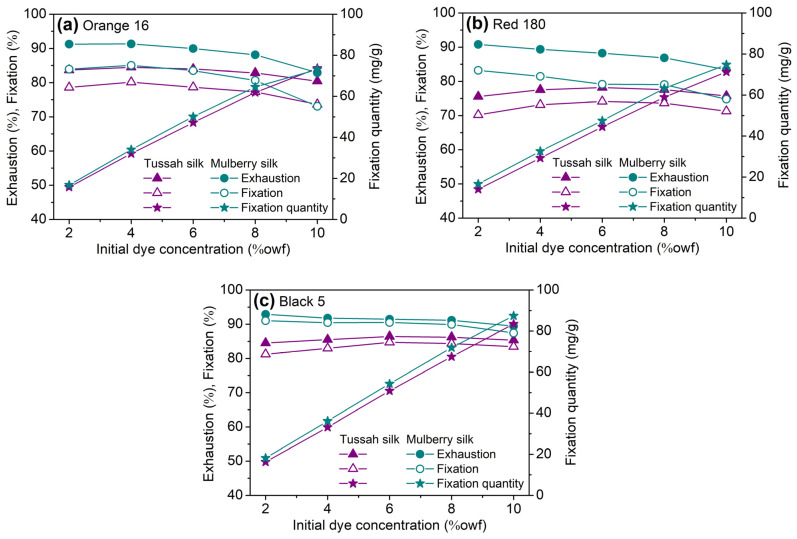
Build-up properties of Orange 16 (**a**), Red 180 (**b**), and Black 5 (**c**) on tussah and mulberry silks dyed using the temperature-rising procedure described by the exhaustion, fixation and fixation quantity of dyes (2%, 4%, 6%, 8% and 10%owf dye, corresponding to 60, 70, 80, 90, and 90 g/L Na_2_SO_4_, respectively; 1 g/L NaHCO_3_).

In general, a high dye dosage results in low exhaustion and fixation. The dyeing of mulberry silk conformed to this rule, as shown in Figure 6, while the dyeing of tussah silk did not fully obey this rule; this was related to the use of different quantities of sodium sulfate at various dosages of dyes. The exhaustion of Orange 16, Red 180, and Black 5 on tussah silk was higher than 80%, 75%, and 85%, respectively, for all dye dosages. Orange 16 and Red 180 had fixation rates of 77–80% and 73% on tussah silk at dye dosages of 2–8%owf and 4–8%owf, respectively, whereas Black 5 had a fixation rate of over 81% on tussah silk at various dosages. Three SES dyes exhibited a high fixation ability on tussah silk. The fixation quantity of three SES dyes on tussah and mulberry silks increased almost linearly with an increase in dye dosage. In terms of fixation quantity alone, three dyes had good build-up capabilities on tussah and mulberry silks.

As shown in Figure 6, at almost all dye dosages and under the present alkaline fixation conditions, the exhaustion, fixation, and fixation quantity of these three dyes on tussah silk were lower than on mulberry silk. Such dyeing results were unexpected, given the difference between the reactive amino acid content of tussah and mulberry silks. Although the content of tyrosine in tussah silk is slightly lower than that in mulberry silk (10.60% vs. 11.29%wt), the content of basic amino acids in tussah silk is significantly higher than that in mulberry silk (7.13%wt vs. 1.84%wt) [24]. The lower exhaustion, fixation, and fixation quantity of reactive dyes on tussah silk are very likely related to the following factors: compared with mulberry silk, tussah silk has a higher content of acidic amino acids, generating more negative charges [24], smaller surface area [30,31], and lower swelling [32,33], all of which decrease the adsorption quantity of dyes. However, on the whole, the fixation efficiency of SES dyes on tussah silk was slightly higher than that on mulberry silk, which was evidenced by the smaller difference between the exhaustion and fixation of tussah silk, as depicted in Figure 6. This phenomenon is associated with the complete reaction between the VS groups of dyes and tussah silk with a greater number of amine groups.

**Figure 7:**
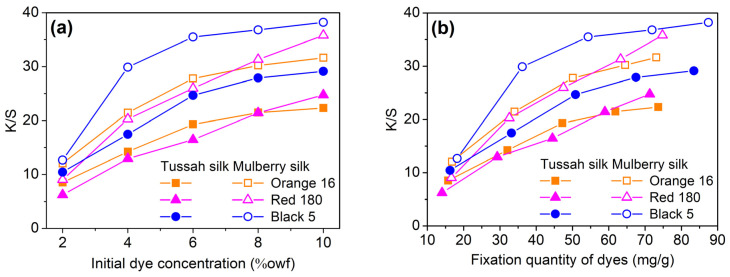
The build-up properties of SES dyes on tussah and mulberry silks dyed using the temperature-rising procedure described in terms of the apparent color depth: (**a**) K/S versus initial dye dosage, and (**b**) K/S versus dye fixation quantity.

As shown in Figure 7, the apparent color depth (K/S) of dyed tussah and mulberry silks both increased as the initial dosage and fixation quantity of dye increased, indicating the good build-up capability of SES dyes on the two silks. However, the increment in the color depth of dyed tussah silk with increasing dosage of dyes was smaller, compared to that observed for mulberry silk. In other words, from the perspective of apparent color depth, the build-up capability of SES dyes on tussah silk was inferior to that on mulberry silk.

**Figure 8:**
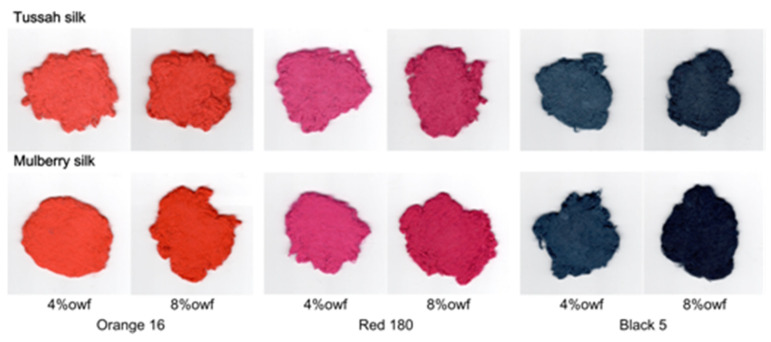
Images of tussah and mulberry silks dyed with 4% and 8%owf Orange 16, Red 180, and Black 5.

As shown in Figure 7, as both the dye dosage and the dye fixation quantity were the same, the apparent color depth of tussah silk was significantly lower than that of mulberry silk. This was confirmed by the visual images of dyed samples shown in Figure 8. In other words, in the case of reactive dyeing, tussah silk exhibited poorer color rendering performance than mulberry silk. To achieve the same apparent color depth as mulberry silk, tussah silk requires a higher amount of dosage and fixation of reactive dyes.

**Figure 9:**
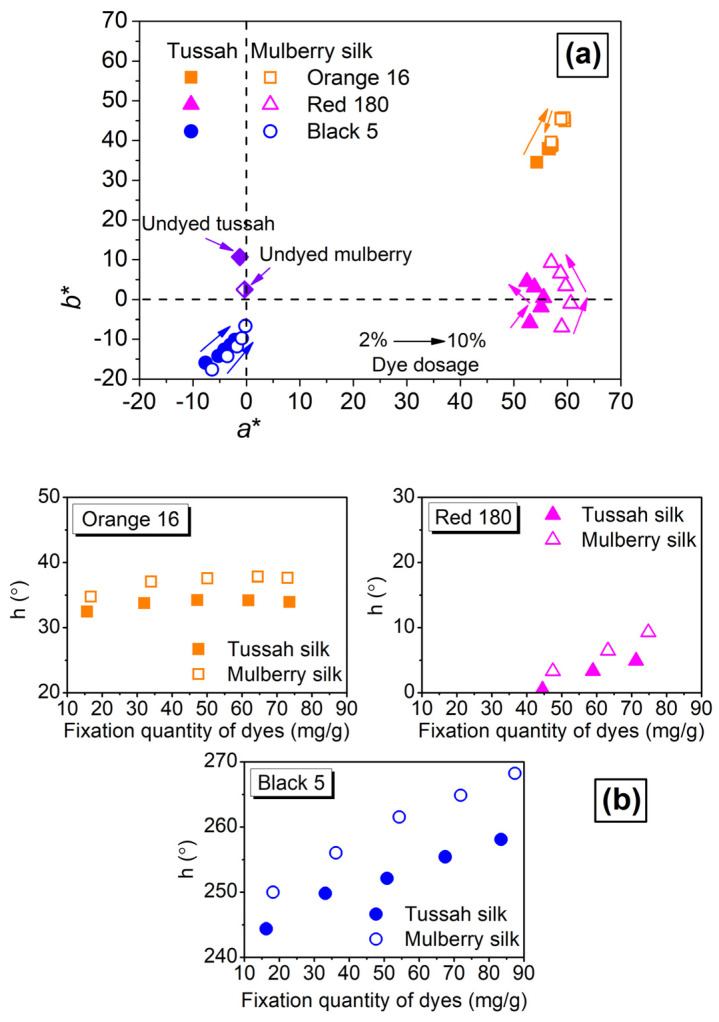
Chromaticity diagram (**a**) and hue angle (**b**) of tussah and mulberry silks dyed at various dosages of SES dyes.

From Figure 9, it can be seen that the chromaticity points of tussah silk dyed with Orange 16 and Red 180 were more located on the inner side of the chromaticity diagram compared to dyed mulberry silk, indicating that dyed tussah silk has lower color saturation or brilliance than dyed mulberry silk. Moreover, the hue angles of dyed tussah and mulberry silks were also inconsistent. As the number of fixed dyes increased, the difference in hue angle between the two silks increased; this was especially true for the Black 5 dyed silks. It was preliminarily speculated that the difference in color brilliance between the two silks was related to the distribution of reactive dyes in the fiber interior (e.g., aggregation of dyes in the macro-voids of tussah silk) and the low whiteness of tussah silk caused by trace amounts of natural pigments.

#### 2.2.5. Effect of Liquor Ratio

In current production, the liquor ratio of dyeing is mainly decided by the dyeing machines used. For the dyeing of silk products, various types of machines are employed, depending on the forms of silk fibers (e.g., loose fibers, yarns, and fabrics) and the textures of fabrics (e.g., plain and crepe). Generally, the liquor ratio used in silk dyeing is higher than that in the dyeing of other fibers in order to avoid or decrease the friction damage of silk fibers or fabrics, because silk is susceptible to mechanical forces and friction. Therefore, it is necessary to explore the effect of the liquor ratio on the dyeing of tussah silk. In the present study, a high liquor ratio was used to ensure the color uniformity of loose tussah silk, which is much higher than that used in widely applied dyeing processes.

In our test of the liquor ratio, a sample dyeing machine with infrared heating was used, and two dyes with different substantivities were purposely selected. Red 180 is a monoazo dye with three sulfonate groups and one SES group, while Black 5 is a diazo dye with two sulfonate groups and two SES groups. The latter has higher substantivity than the former. It can be predicted that when the liquor ratio changes, the dye with higher substantivity will have smaller changes in its exhaustion and fixation. In other words, the use of a low liquor ratio is more conducive to the improvement in the exhaustion and fixation of Red 180 with low substantivity.

**Figure 10:**
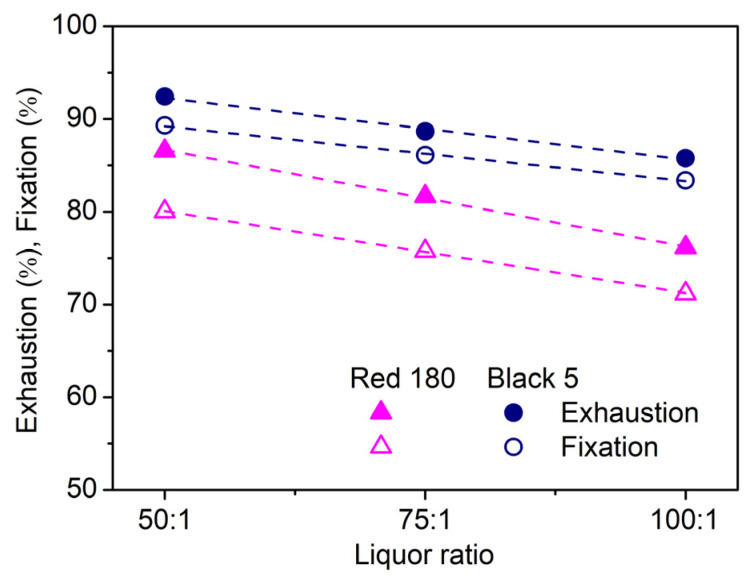
Influence of liquor ratio on the exhaustion and fixation of Red 180 and Black 5 on tussah silk dyed in an infrared dyeing machine (4%owf dye, 700%owf Na_2_SO_4_, and 10%owf NaHCO_3_).

In this test, three liquor ratios (100:1, 75:1, and 50:1) were employed, and the dosage of dyes and chemicals was based on the relative fiber weight to ensure that the same absolute amount of all chemicals would present in the dye solution. As shown in Figure 10, with a decrease in liquor ratio, the exhaustion and fixation of two dyes displayed a linear increasing trend (regression coefficient > 0.99). This means that if the results of this study were applied to the low liquor ratio used in current production practice, the fixation of dyes would be significantly improved. In addition, from Figure 10, it can be seen that the slope of the linear regression line of Red 180 was larger, indicating that the exhaustion and fixation of Red 180 with low substantivity increased more significantly with a decreasing liquor ratio.

#### 2.2.6. Color Fastness to Soaping

Four SES dyes (Orange 16, Red 180, Blue 19, and Black 5) (Black 5, Bis(SES)) at 4%owf were used to dye tussah silk. The dyeing process included the following steps: elevating temperature from 30 °C to 90 °C, holding the temperature for fixation at 90 °C, washing, soaping twice, and washing. Two fixation methods were used, i.e., fixation in an alkaline medium (1 g/L sodium bicarbonate) for 60 min and fixation in an alkali-free medium for 90 min. The exhaustion, fixation, fixation efficiency, and soaping color fastness of SES dyes are shown in Table 4.

As shown in Table 4, when sodium bicarbonate was used as a fixing agent at 90 °C, Orange 16, Blue 19, and Black 5 exhibited higher exhaustion, fixation, and fixation efficiency compared with fixation under a neutral condition at 90 °C for a long time. This was closely related to the fact that the VS groups of dyes, which were converted from SES groups under alkaline conditions, could react with both the amine and hydroxyl groups in tussah silk. Figure S4 shows that the samples dyed with Orange 16 and Blue 19 in the presence of an alkali exhibited increased apparent color depth compared to those dyed in the absence of an alkali.

In this experiment, the dyed tussah samples obtained by two fixing methods and soaping twice exhibited excellent color fastness to soaping (color change and staining), i.e., a 4–5 rating or higher. Figure S4 also reveals that the dyed samples showed almost no discoloration or fading after soaping. Therefore, the application of SES dyes can solve the existing problem of poor soaping fastness for the dyeing of tussah silk with acid dyes, 1:2 metal complex dyes, and direct dyes [3].

### 2.3. Dyeing of Tussah Silk with SES Dyes under Neutral Conditions

#### 2.3.1. Comparison of the Boiling Dyeing of Tussah and Mulberry Silks

**Figure 11:**
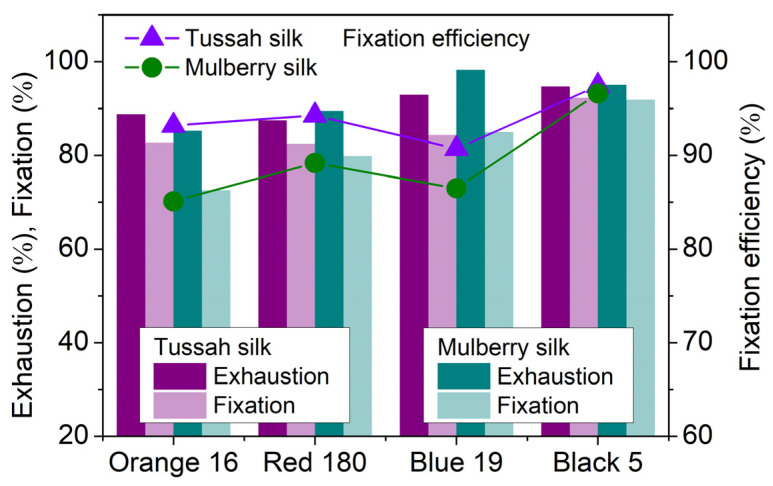
Exhaustion, fixation, and fixation efficiency of Orange 16, Red 180, Blue 19, and Black 19 on tussah and mulberry silks dyed at 100 °C (4%owf dye and 70 g/L Na_2_SO_4_; temperature-rising dyeing; holding time at 100 °C, 90 min).

Table 4 shows that the alkaline dyeing of SES dyes at 90 °C has distinct advantages over neutral dyeing at 90 °C in terms of exhaustion, fixation, and fixation efficiency, because of the reaction of both amine and hydroxyl groups in tussah silk with VS groups in dyes. Taking into account that increasing the temperature can accelerate the conversion of SES groups into VS groups and improve the swelling extent of tussah silk, the boiling dyeing of tussah silk at 100 °C under neutral conditions was attempted in order to increase the exhaustion and fixation of SES dyes. The results are shown in Figure 11.

Compared with neutral dyeing at 90 °C in Table 4, boiling dyeing greatly improved the exhaustion and fixation of the four SES dyes. The exhaustion and fixation of Orange 16, Red 180, Blue 19, and Black 5 (Black 5, Bis(SES)) on tussah silk increased to 88.77% and 82.72%, 87.46% and 82.45%, 92.95% and 84.32%, 94.71%, and 92.26%, respectively. This significant increase in exhaustion and fixation validates the high conversion of SES groups into VS groups at 100 ℃ and under neutral conditions. On the other hand, the fixation of Orange 16, Red 180, and Black 5 on tussah silk in neutral boiling dyeing exceeded that in alkaline dyeing at 90 °C, while the fixation of Blue 19 was almost the same as that in alkaline dyeing at 90 °C. Importantly, in neutral boiling dyeing, the fixation of Blue 19 and Black 5 on tussah silk was almost identical to that on mulberry silk, while the fixation of Orange 16 and Red 180 on tussah silk was higher than that on mulberry silk, as shown in Figure 11. Therefore, neutral boiling dyeing offers the major advantage of high dye fixation.

Furthermore, Figure 11 also shows that in neutral boiling dyeing, SES dyes had higher fixation efficiency on tussah silk than on mulberry silk; this can be explained by the presence of more reactive groups in tussah silk. Additionally, the fixation efficiency values of four SES dyes with an average of 93.90% were very close to those in alkaline dyeing at 90 °C, with an average of 94.76%. As a consequence, neutral boiling dyeing has another advantage of high dye fixation efficiency, which can reduce the burden of washing and soaping after dyeing.

#### 2.3.2. Effect of Holding Time for Boiling Dyeing

Figure 5 reveals the gradual increase in the fixation of Red 180 during neutral dyeing at 90 °C due to the slow conversion of SES groups to VS groups, in contrast to the rapid increase observed under alkaline fixation. Considering the slow fixation of SES dyes, it is necessary to explore the effect of holding time in neutral boiling dyeing.

**Figure 12:**
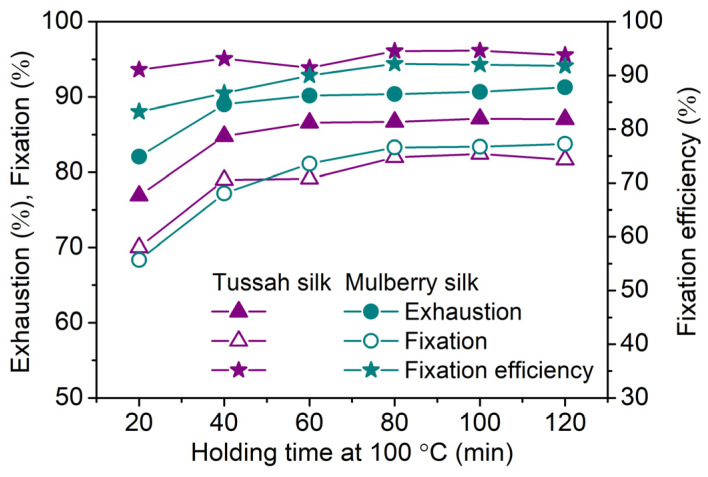
Effect of holding time at 100 °C on the exhaustion, fixation, and fixation efficiency of Red 180 on tussah silk (3%owf dye and 80 g/L Na_2_SO_4_; temperature-rising dyeing).

As shown in Figure 12, in neutral boiling dyeing, the exhaustion and fixation of Red 180 on tussah and mulberry silks increased gradually and tended to balance when the holding time reached 60–80 min. However, in neutral dyeing at 90 °C (Figure 5), exhaustion and fixation equilibrium did not occur after dyeing for 90 min at this temperature. This revealed that for neutral dyeing, increasing the temperature from 90 °C to 100 °C can significantly speed up the conversion of SES groups to VS groups and thus facilitate the reaction between SES dyes and silk fibers. In alkaline fixing at 90 °C (Figure 5), the fixation quantity reached the maximum after sodium bicarbonate was added for 15 min. Compared with alkaline fixation, it was evident that neutral boiling dyeing required a longer holding time, which is one drawback. However, due to the gradual increase in dye fixation, neutral boiling dyeing has the big advantage of good dyeing levelness.

#### 2.3.3. Color Fastness to Soaping

The dyed tussah samples in Figure 11 were subjected to the test of color fastness to soaping. As shown in Table 5, the fastness to soaping of dyed tussah silk obtained by neutral boiling dyeing was as good as that of dyed tussah silk obtained by alkaline fixing at 90 °C, i.e., a 4–5 rating or higher. The fastness test also indicated that the neutral boiling dyeing of tussah silk is completely feasible.

## 3. Materials and Methods

### 3.1. Materials

Degummed and bleached loose tussah and mulberry silk fibers were bought from Taihu Lake Snow Co., Ltd., Suzhou, China. Four different types of reactive dyes were used, i.e., sulfatoethylsulfone (SES), sulfatoethylsulfone/monochlorotriazine (SES/MCT), monochlorotriazine (MCT), and bis(monochlorotriazine) (Bis(MCT)) dyes. Three SES dyes (C.I. Orange 16, Red 180, and Black 5) (Black 5, Bis(SES)) and MCT dyes were purchased from Meryer (Shanghai) Biochemical Technology Co., Ltd., Shanghai, China. Bis(MCT) dyes were kindly provided by Everlight Chemical Industrial Co., Taipei, Taiwan, China. SES/MCT dyes and C.I. Reactive Blue 19 were provided by Taixing Zhenqing Chemical Co., Ltd., Taixing, China. Leveler O (fatty alcohol polyoxyethylene ether, nonionic surfactant) was obtained from Jiangsu Haian Petrochemical Plant, Haian, China, and Soaping Agent 506G was a commercial product from a local dyeing mill. Sodium sulfate, sodium carbonate, sodium bicarbonate, and sodium hydroxide were of analytical grade.

### 3.2. Dyeing Methods

Most of the dyeing experiments were carried out in a XW-ZDR-25X12 sample dyeing machine with water-bath heating and oscillating functions (Jingjiang Xinwang Dyeing and Finishing Equipment Factory, Jingjiang, China), and a small portion of dyeing was conducted in a FAD-7-18P infrared heating dyeing machine (Wuxi Kelinan Technology Co., Ltd., Wuxi, China). In all dye baths, 0.4 g/L Leveler O was added. Unless otherwise stated, the XW-ZDR-25X12 sample dyeing machine and a bath ratio of 100:1 were used.

#### 3.2.1. Reactive Dyeing under Alkaline Conditions

The experiments on the effects of temperature and alkaline agents on dyeing with SES/MCT, SES, MCT, and Bis(MCT) dyes, and the effects of sodium sulfate and sodium bicarbonate dosage on dyeing with SES dyes were conducted at constant temperatures. The dyeing was firstly carried out in the presence of sodium sulfate for 40 min, and then alkaline agents were added, and the alkaline fixing was performed for 60 min. Finally, dyed samples were subjected to complete washing in distilled water and soaping at 90 °C for 20 min in a solution of 2 g/L Soaping Agent 506G. Other details were as follows:

To assess the effects of temperature and alkaline agents, 3%owf dye, 60 g/L sodium sulfate, and 3 g/L sodium carbonate or sodium bicarbonate were used, and dyeing temperatures ranging from 50 °C and 90 °C were set. In the sodium sulfate experiment, 5%owf dye, 30–105 g/L sodium sulfate, and 3 g/L sodium bicarbonate were used, and the dyeing and fixing temperatures were 90 °C. In the sodium bicarbonate dosage experiment, 2.5%owf dye vs. 60 g/L sodium sulfate, 5.0%owf dye vs. 90 g/L sodium sulfate, and 0–6 g/L sodium bicarbonate were used, and the dyeing and fixing temperatures were 90 °C.

The experiments regarding the exhaustion and fixation kinetics of SES dyes, the build-up properties of SES dyes, and the preparation of tussah samples dyed with SES dyes for the color fastness evaluation were carried out using the temperature-rising procedure. Dyeing was started at 30 °C with the addition of sodium sulfate. The dye solution was then heated to 90 °C at 1 °C/min. After sodium bicarbonate had been added at 90 °C, dyeing was continued for the required times. Finally, dyed samples were subjected to sufficient washing in distilled water and double soaping at 90 °C. Other details were as follows:

To assess the exhaustion and fixation kinetics, 3%owf dye, 80 g/L sodium sulfate, and 0 or 2 g/L sodium bicarbonate were employed, and the holding time at 90 °C was 90 min. In the experiment of the build-up ability of SES dyes, 2–10%owf dye, 60–90 g/L sodium sulfate, and 1 g/L sodium bicarbonate were employed; when the dye dosage was 2%, 4%, 6%, 8%, and 10%owf, the dosage of sodium sulfate was 60, 70, 80, 90, and 90 g/L, respectively; the holding time at 90 °C was 60 min. To prepare the dyed tussah samples for our color fastness evaluation, 4%owf dye, 70 g/L sodium sulfate, and 0 or 1 g/L sodium bicarbonate were used; the holding time at 90 °C was 60 min for the alkaline fixing and 90 min for the neutral fixing.

To examine the effect of the liquor ratio on the exhaustion and fixation of SES dyes, three liquor ratios (100:1, 75:1, and 50:1) were used and the dyeing was implemented in an infrared heating dyeing machine using the temperature-rising procedure. In this test, the concentration of dyes and chemicals was determined based on the relative fiber weight to ensure that the same absolute quantity of all chemicals would be present in the dye baths with various liquor ratios. The formulation of the dye solution was as follows: 4%owf dye, 700%owf sodium sulfate, 10%owf sodium bicarbonate, and 4%owf Leveler O (equivalent to 4%owf dye, 70 g/L sodium sulfate, 1 g/L sodium bicarbonate, and 0.4 g/L Leveler O at a liquor ratio of 100:1). The dyeing was started at 30 °C in the presence of sodium sulfate, and the dye solution was heated to 90 °C at 1 °C/min. After the addition of sodium bicarbonate at 90 °C, dyeing was continued for 60 min. Finally, the dyed samples were subjected to washing and double soaping.

#### 3.2.2. Reactive Dyeing under Neutral Conditions

The dyeing was implemented in the infrared heating dyeing machine using the temperature-rising procedure. Dyeing was started at 30 °C in the presence of sodium sulfate. The solution of SES dyes was heated to 100 °C at 1 °C/min, and at this temperature, the dyeing was continued for the required times. Finally, the dyed samples were subjected to washing and double soaping. In our experiment of the effect of holding time at 100 °C, 3%owf dye and 80 g/L sodium sulfate were employed, and the holding time was set in the range of 20–120 min. For our comparative study of the exhaustion and fixation of SES dyes on tussah and mulberry silks, 4%owf dye, 70 g/L sodium sulfate, and a holding time of 90 min were employed. The resulting tussah samples were then subjected to the test of color fastness to soaping.

### 3.3. Measurements

The exhaustion percentage of reactive dyes was determined based on the colorimetry of the residual dyebath. The absorbance of the dye solution was measured using a UV-1800 UV–visible spectrophotometer (Shimadzu Co., Kyoto, Japan). The percentage of exhaustion was calculated using Equation (1):

(1) $$\mathrm{Exhaustion}\;(\%)=\frac{A_{0}-A_{1}}{A_{0}}\times 100$$

where *A*_0_ and *A*_1_ represent the absorbance of the dye solution before and after dyeing, respectively.

The percentage of dye fixation was calculated using Equation (2):

(2) $$\mathrm{Fixation}\;(\%)=\frac{A_{0}-A_{1}-A_{2}}{A_{0}}\times 100$$

where *A*_2_ represents the absorbance of the soaping solution.

The fixation efficiency of reactive dyes was calculated using Equation (3):

(3) $$\mathrm{Fixation}\;\mathrm{efficiency}\;(\%)=\frac{Fixation}{Exhaustion}\times 100$$

The apparent color depth (*K*/*S*), red–green index (a*), yellow–blue index (b*), and hue angle (*h*) of the dyed samples were measured on an UltraScan PRO reflectance spectrophotometer (Hunter Associates Laboratory Inc., Reston, VA, USA) using a D65 light source and a 10° standard observation angle. Each sample was tested at five different positions, and the average value was taken.

Color fastness to soaping was tested according to GB/T 3921-2008 Test Method A on a WASH TEC-P fastness tester (Roaches International, West Yorkshire, UK).

## 4. Conclusions

SES, SES/MCT, MCT, and Bis(MCT) dyes were used to dye tussah silk. The dyeing temperature and alkaline fixation tests demonstrated that SES dyes were more suitable for the dyeing of tussah silk. SES dyes showed a high degree of fixation on tussah silk when dyeing was carried out at 90 °C and with the use of sodium bicarbonate as an alkaline agent. The fixation of four SES dyes (Orange 16, Red 180, Blue 19, and Black 5) on tussah silk ranged from 70% and 85%, with a fixation efficiency of above 90%, i.e., approximately 10% lower than that on mulberry silk.

Sodium sulfate (dyeing accelerator) dosage, sodium bicarbonate (fixing agent) dosage, and the liquor ratio had significant impacts on the dyeing with SES dyes. The dyeing of tussah silk was more sensitive to sodium sulfate dosage compared to that of mulberry silk, and it required more neutral electrolytes. Dyes with more sulfonate groups and lower substantivity were more sensitive to the sodium sulfate dosage and liquor ratio. The dyeing of tussah silk with SES dyes at 90 °C only required a very small amount of sodium bicarbonate (e.g., 1 g/L) to achieve a high degree of fixation. The SES dyes used in alkaline dyeing showed the advantages of high fixation, high fixation efficiency, time-saving, excellent soaping color fastness, and ability to react with both amine and hydroxyl groups in tussah silk; however, they also had the shortcoming of rapid fixation, leading to uneven dyeing. Under alkaline dyeing conditions and at 90 °C, SES dyes showed good build-up on tussah silk. However, the apparent color depth and color brilliance of dyed tussah silk were lower than those of dyed mulberry silk, and tussah silk required a higher dosage of dyes to achieve the same color depth as mulberry silk.

SES dyes could be also applied in the dyeing of tussah silk at 100 °C and under neutral conditions with the use of sodium sulfate as an accelerator. Neutral boiling dyeing with SES dyes and tussah silk exhibited higher exhaustion and fixation, roughly equal fixation efficiency, slower fixation speed, and better color levelness, compared with the alkaline dyeing at 90 °C. Interestingly, in neutral boiling dyeing, SES dyes displayed higher and more efficient fixation on tussah silk than on mulberry silk. However, neutral boiling dyeing required a longer fixation time than alkaline dyeing at 90 °C due to the slow conversion of SES groups to VS groups. All dyed tussah samples showed excellent color fastness to soaping.

## Figures and Tables

**Table 1:** Dyes, dyeing conditions, and results regarding the reactive dyeing of tussah silk reported previously.

Reactive Dye	Depth (%owf)	Temperature (°C)	Accelerant Alkali	Fixation (%)	Reference
*Sulfatoethylsulfone/monochlorotriazine dye (SES/MCT)*					
Reactive Red M-3BE	2, 8	80	Na_2_SO_4_ 60 g/L NaHCO_3_ 2.5 g/L	37%	[9]
Reactive Yellow B-3BD, Red B-2BF and Blue B-KV	1	60	Na_2_SO_4_ 20 g/L Na_2_CO_3_ 15 g/L	60–65%	[10]
Yellow B-4RFN and Red B-2BF	–	60	Na_2_SO_4_ 40 g/L Na_2_CO_3_ + NaHCO_3_ 15 g/L	55–65%	[11]
*Bis(monochlorotriazine) dye (Bis(MCT))*					
Reactive Yellow KE-3G, Red KE-3B, and Blue KE-R	1	85	CH_3_COOH (pH 4.6) CH_3_COOH (pH 4.1)	56–66% 57–77%	[12]
*Mononicotinic acid triazine dye (MNT)*					
Kayacelon React Red CN-3B and Blue CN-BL	1–2	99	Na_2_HPO_4_ + NaH_2_PO_4_ 2 g/L (pH 6.5–7.5) Na_2_SO_4_ 20–80 g/L	60–75%	[13]
*Dichlorotriazine dye (DCT)*					
Reactive Brilliant Red X-3B	–	Room temperature	NaCl 40 g/L Na_2_CO_3_ 10 g/L	41%	[14]

**Table 2:** Content of alkaline and hydroxy amino acids present in tussah and mulberry silks [24,25].

Amino Acid		Content (%wt)		
		Tussah Silk	Mulberry Silk	Wool
*Hydroxy amino acids*				
Serine	Ser	14.80	15.98	9.04
Tyrosine	Tyr	10.60	11.29	6.38
Threonine	Thr	0.20	1.49	6.55
Total		25.60	28.76	21.97
*Alkaline amino acids*				
Lysine	Lys	0.17	0.56	2.82
Argnine	Arg	5.41	0.98	10.49
Histidine	His	1.55	0.30	0.90
Total		7.13	1.84	14.21

**Table 3:** Reactive dyes used.

C.I. Reactive	Trade Name	Chromophore	Molecular Mass (g/mol)	Number of –SO_3_Na
*Sulfatoethylsulfone dye (SES)*				
Orange 16	Reactive Orange 16	Monoazo	617.5	1
Red 180	Reactive Red 180	Monoazo	933.8	3
Black 5	Reactive Black 5 (Bis(SES))	Bisazo	991.8	2
Blue 19	Starzol Brill. Blue KN-RHG	Anthraquinone	626.6	1
*Monochlorotriazine dye (MCT)*				
Yellow 3	Reactive Yellow 3	Monoazo	637.0	2
Red 24	Reactive Red 24	Monoazo	788.1	3
Blue 13	Procion Blue H-5R	Monoazo (1:1 Cu complex)	942.2	4
*Sulfatoethylsulfone/monochlorotriazine dye (SES/MCT)*				
Yellow 160	Starzol Brill. Yellow LB-4GLN	–	–	–
–	Starzol Yellow LB-3RD	Monoazo	–	–
Yellow 145	Starzol Yellow LB-4RFN	Monoazo	1026.3	3
–	Starzol Scarlet LB-3G	Monoazo	–	–
Red 195	Starzol Red LB-3BF	Monoazo	1136.3	4
Blue 194	Starzol Dark Blue LB-2GLN	Bisazo	1205.4	4
Blue 231	Starzol Turquoise Blue LB-BGFN	Phthalocyanine	–	–
–	Starzol Black LB-ED	Azo	–	–
*Bis(monochlorotriazine) dye (Bis(MCT))*				
Orange 84	Evercion Orange H-ER	Bisazo	1850.3	8
Red 141	Evercion Red H-E7B	Bisazo	1774.2	8
Blue 171	Evercion Navy Blue H-ER	Bisazo	1418.9	6

**Table 4:** Soaping color fastness of tussah silk dyed with 4%owf SES dyes at 90 °C.

Dyeing Method	Dye	Exhaustion (%)	Fixation (%)	Fixation Efficiency (%)	Fastness		
					Color Change	Staining	
						Silk	Cotton
Alkali (NaHCO_3_)	Oange 16	84.37	79.61	94.36	5	4–5	4–5
	Red 180	77.39	72.59	93.80	4–5	5	4–5
	Blue 19	91.54	85.19	93.06	4–5	5	5
	Black 5	84.65	82.80	97.81	4–5	4–5	5
No alkali	Oange 16	66.93	58.70	87.70	5	5	4–5
	Red 180	78.87	73.22	92.84	4–5	4–5	4–5
	Blue 19	77.97	65.21	83.63	4–5	4–5	5
	Black 5	82.99	78.27	94.31	4–5	4–5	5

**Table 5:** Soaping color fastness of tussah silk dyed with 4%owf SES dyes at 100 °C.

Dye	Color Change	Staining	
		Silk	Cotton
Oange 16	5	5	4–5
Red 180	4–5	4–5	4–5
Blue 19	5	4–5	5
Black 5	4–5	4–5	5

## Data Availability

The data are available from the corresponding author upon reasonable request. The data are not publicly available due to limitations in the length of the paper.

## Supplementary Materials

The following supporting information can be downloaded at: https://www.mdpi.com/article/10.3390/molecules29051151/s1, Figure S1: Chemical structures of SES, MCT, SES/MCT, and Bis(MCT) dyes; Figure S2: Exhaustion and fixation of SES/MCT dyes on tussah and mulberry silks dyed at various constant temperatures with the addition of an alkali (dye 3%owf, 60 g/L Na_2_SO_4_, and 3 g/L Na_2_CO_3_ or NaHCO_3_; soaping at 90 °C for 20 min); Figure S3: Exhaustion and fixation of SES, MCT and Bis(MCT) red dyes on tussah and mulberry silks dyed at various constant temperatures with the addition of an alkali (3%owf dye, 60 g/L Na_2_SO_4_, and 3 g/L Na_2_CO_3_ or NaHCO_3_; soaping at 90 °C for 20 min); Figure S4: Images of dyed tussah silk samples before and after the soaping color fastness test (dyeing was carried out with 4%owf SES dyes at 90 °C in the presence and absence of NaHCO_3_).

## Abbreviations

The following abbreviations are used in this manuscript:

*a**	Redness/greenness index
*b**	Yellowness/blueness index
*h*	Hue angle
*K*/*S*	Apparent color depth
MCT	Monochlorotriazine
Bis(MCT)	Bis(monochlorotriazine)
Bis(SES)	Bis(sulfatoethylsulfone)
owf	On the weight of fiber
SES	Sulfatoethylsulfone
SES/MCT	Sulfatoethylsulfone/monochlorotriazine
UV	Ultraviolet
VS	Vinylsulfone
