# Supplementary material for: Dyeing of Tussah Silk with Reactive Dyes: Dye Selection, Dyeing Conditions, Dye Fixation Characteristics, and Comparison with Mulberry Silk
Source: Molecules. 2024 Mar 5;29(5):1151. doi: 10.3390/molecules29051151 (PMC10935422; doi:10.3390/molecules29051151)
Supplement: Supplementary file 1 [file molecules-29-01151-s001.zip › molecules-2895741-supplementary.pdf]

Supplementary Materials

Dyeing of Tussah Silk with Reactive Dyes: Dye Selection, Dyeing Conditions, Dye Fixation Characteristics, and Comparison with Mulberry Silk

Yingjie Yu [1,2,3,4] and Rencheng Tang [1,2,3,4,*]

1 College of Textile and Clothing Engineering, North Campus, Soochow University, Suzhou 215021, China

2 China National Textile and Apparel Council Key Laboratory of Natural Dyes, Dushuhu Campus, Soochow University, Suzhou 215123, China

3 Jiangsu Engineering Research Center of Textile Dyeing and Printing for Energy Conservation, Discharge Reduction and Cleaner Production (ERC), Dushuhu Campus, Soochow University, Suzhou 215123, China

4 National Engineering Laboratory for Modern Silk, Dushuhu Campus, Soochow University, Suzhou 215123, China

* Correspondence: tangrencheng@suda.edu.cn

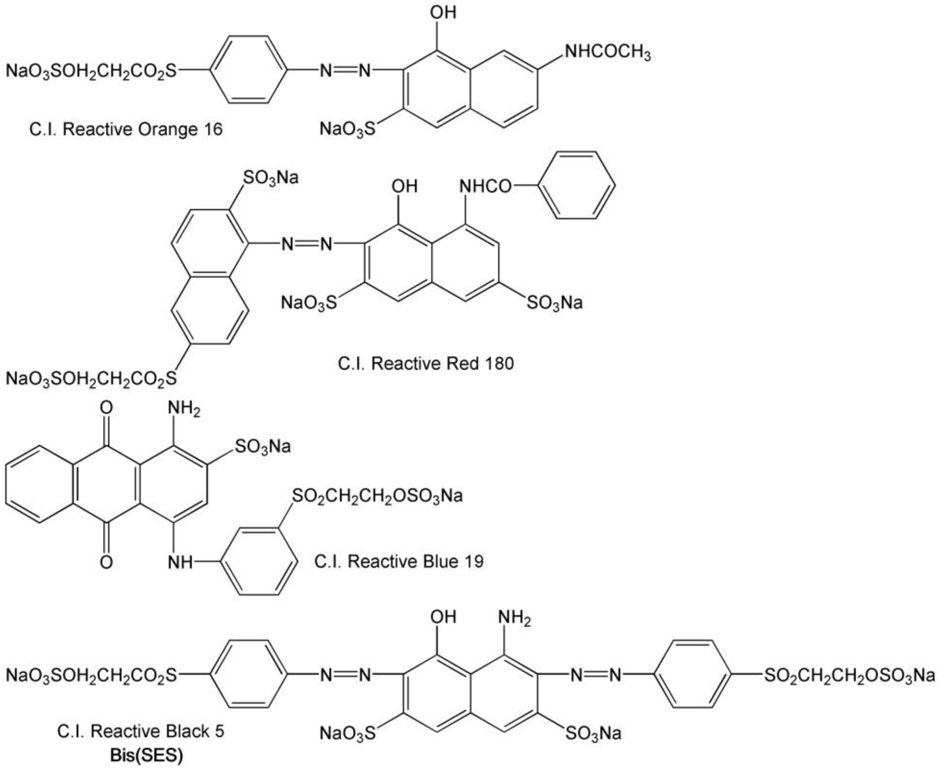

(1) SES dyes

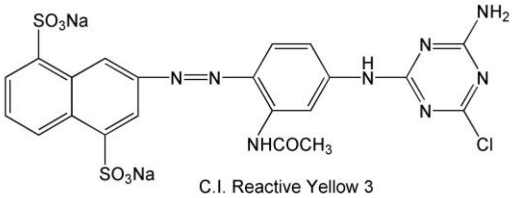

C.I. Reactive Yellow 3

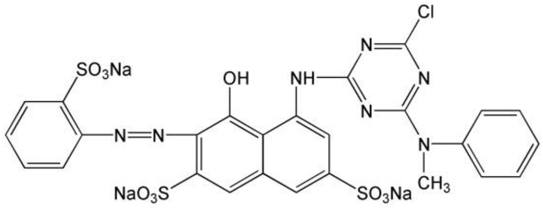

C.I. Reactive Red 24

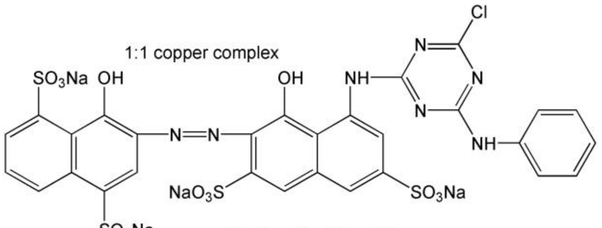

C.I. Reactive Blue 13

(2) MCT dyes

C.I. Reactive Yellow 145

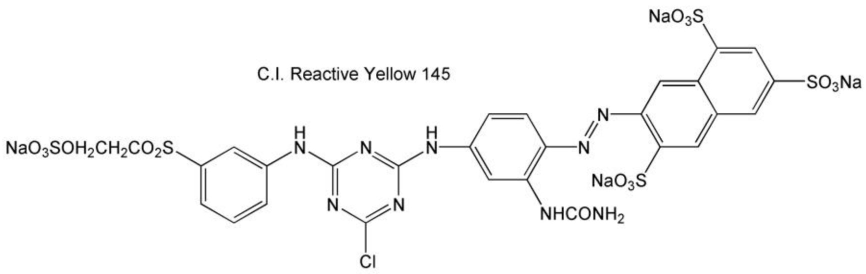

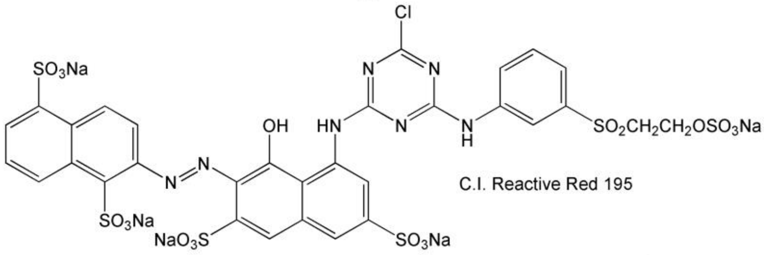

C.I. Reactive Red 195

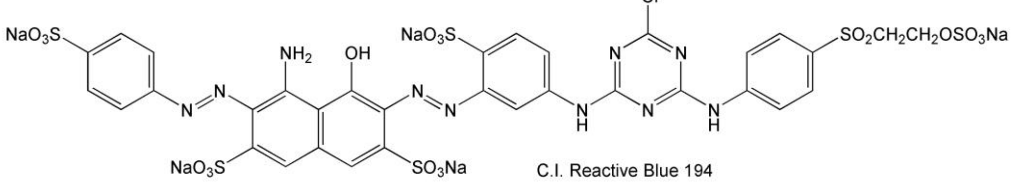

C.I. Reactive Blue 194

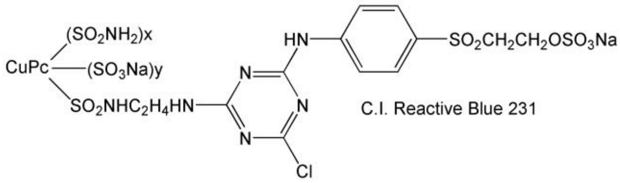

C.I. Reactive Blue 231

(3) SES/MCT dyes

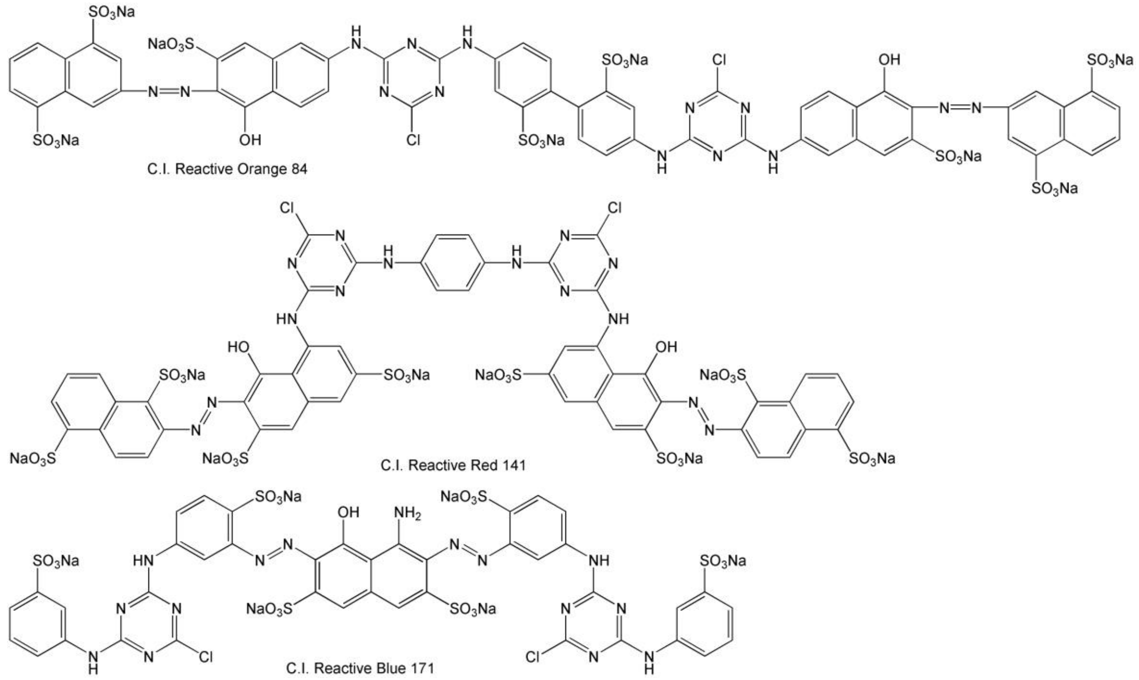

(4) Bis(MCT) dyes

Figure S1. Chemical structures of SES, MCT, SES/MCT, and Bis(MCT) dyes.

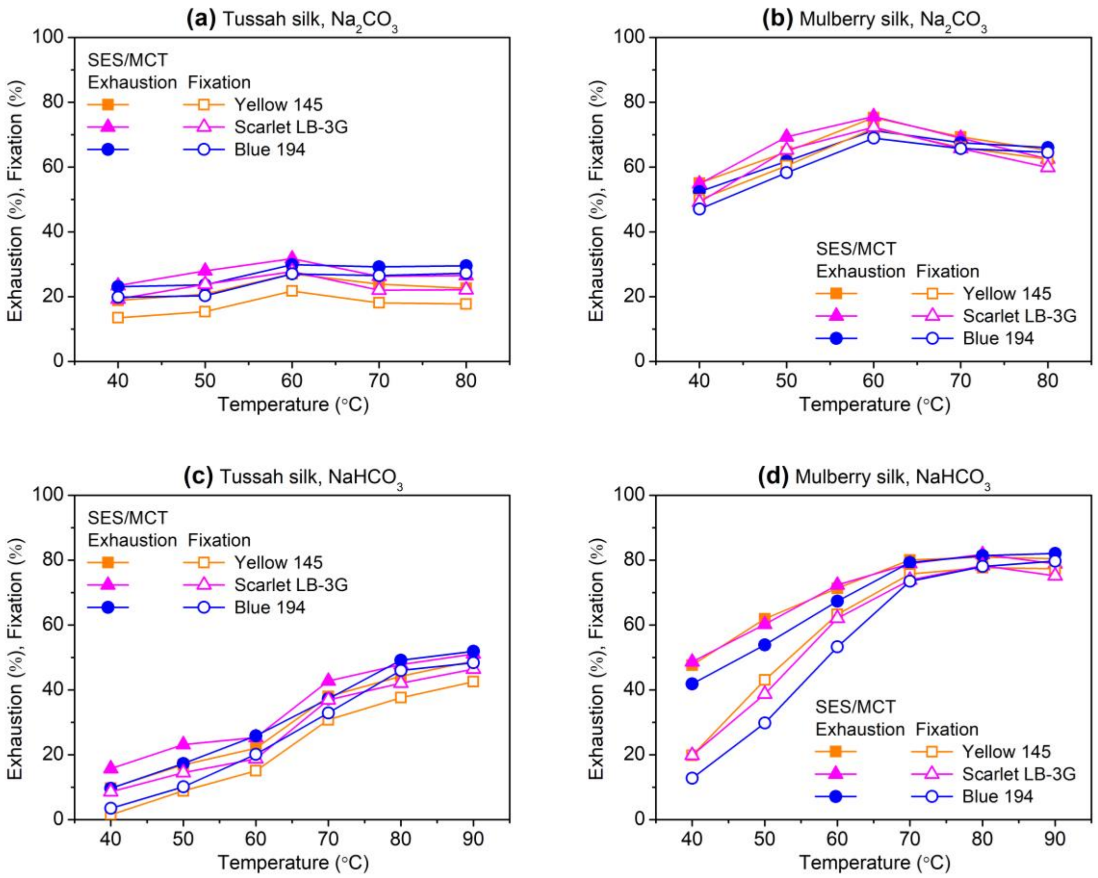

Figure S2. Exhaustion and fixation of SES/MCT dyes on tussah and mulberry silks dyed at various constant temperatures with the addition of an alkali (3%owf dye, 60 g/L Na_2SO_4, and 3 g/L Na_2CO_3 or $NaHCO_3$; soaping at 90 °C for 20 min).

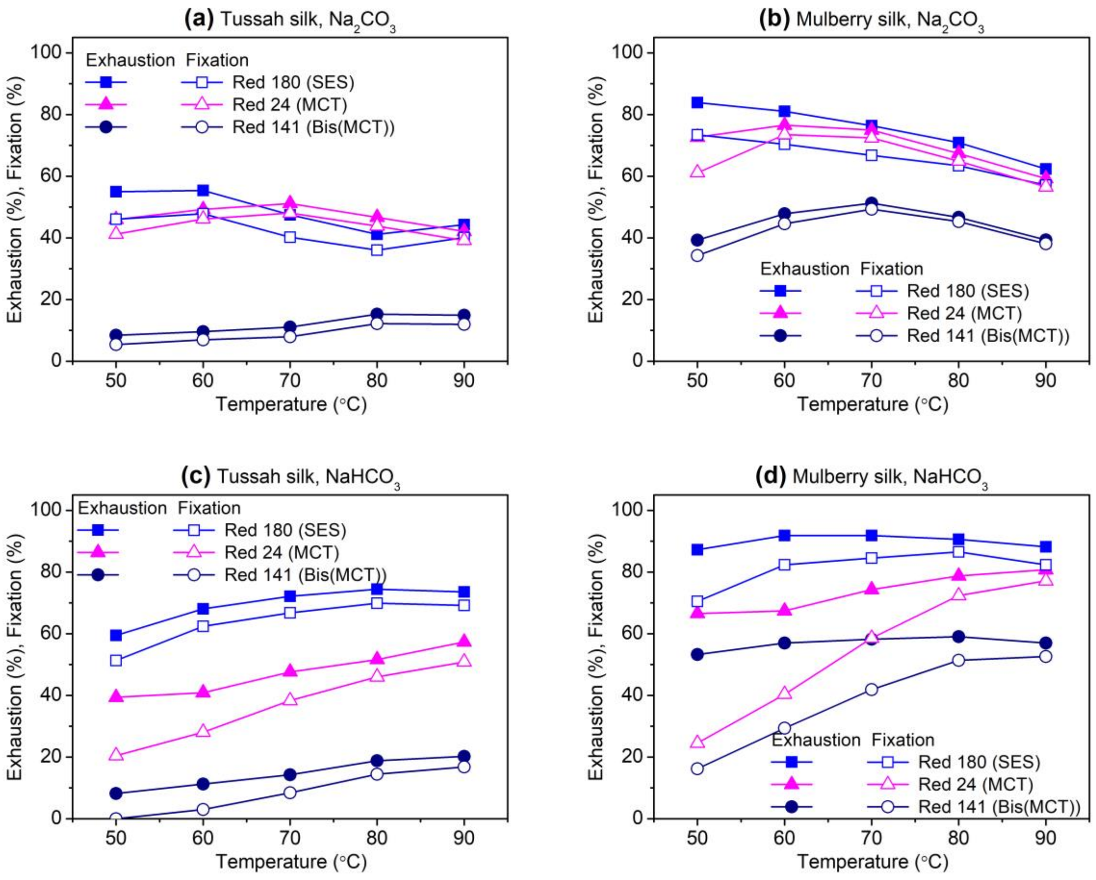

Figure S3. Exhaustion and fixation of SES, MCT, and Bis(MCT) red dyes on tussah and mulberry silks dyed at various constant temperatures with the addition of an alkali (3%owf dye, 60 g/L Na_2SO_4, and 3 g/L Na_2CO_3 or $NaHCO_3$; soaping at 90 ℃ for 20 min).

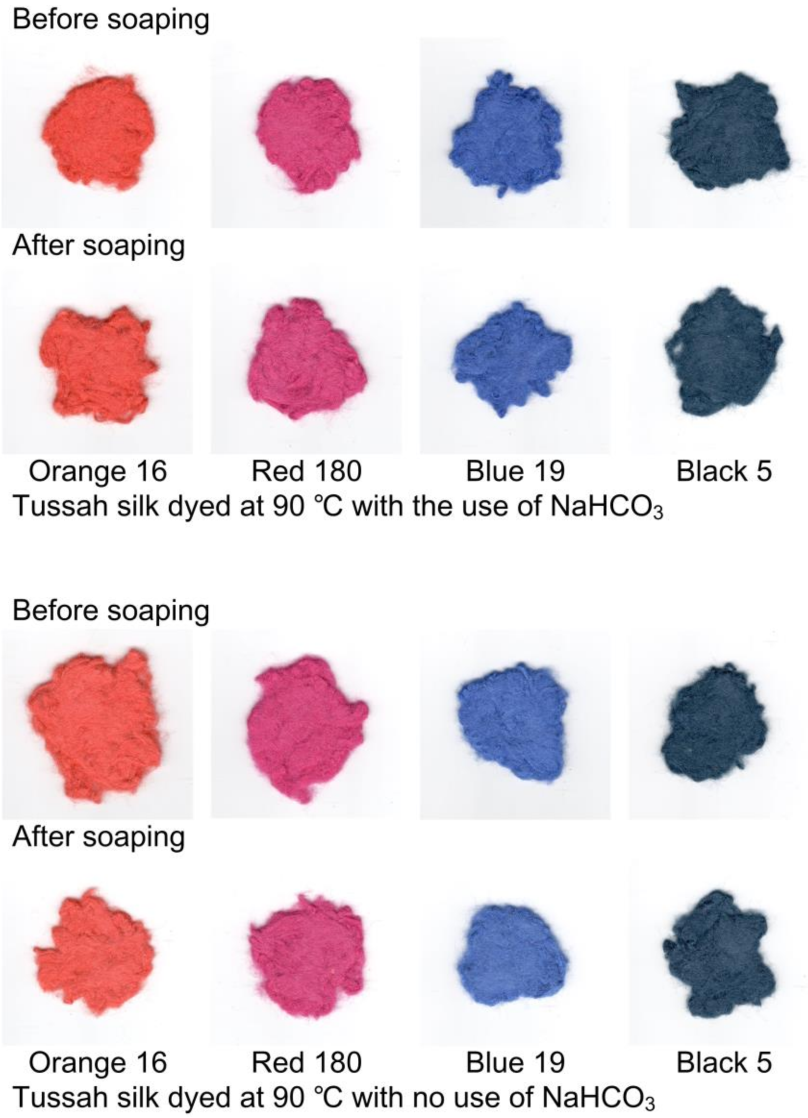

Figure S4. Images of dyed tussah silk samples before and after the soaping color fastness test (dyeing was carried out with 4%owf SES dyes at 90 °C in the presence and absence of $NaHCO_3$)
